# QSAR Modelling to Identify LRRK2 Inhibitors for Parkinson’s Disease

**DOI:** 10.1515/jib-2018-0063

**Published:** 2019-02-14

**Authors:** Víctor Sebastián-Pérez, María Jimena Martínez, Carmen Gil, Nuria Eugenia Campillo, Ana Martínez, Ignacio Ponzoni

**Affiliations:** Centro de Investigaciones Biológicas (CIB-CSIC), Ramiro de Maeztu 9, 28040 Madrid, Spain; Instituto de Ciencias e Ingeniería de la Computación (UNS–CONICET), Departamento de Ciencias e Ingeniería de la Computación, Universidad Nacional del Sur (UNS), Bahía Blanca, Argentina

**Keywords:** Cheminformatics, QSAR, Machine Learning, Parkinson’s disease, LRRK2

## Abstract

Parkinson’s disease is one of the most common neurodegenerative illnesses in older persons and the leucine-rich repeat kinase 2 (LRRK2) is an auspicious target for its pharmacological treatment. In this work, quantitative structure–activity relationship (QSAR) models for identification of putative inhibitors of LRRK2 protein are developed by using an in-house chemical library and several machine learning techniques. The methodology applied in this paper has two steps: first, alternative subsets of molecular descriptors useful for characterizing LRRK2 inhibitors are chosen by a multi-objective feature selection method; secondly, QSAR models are learned by using these subsets and three different strategies for supervised learning. The qualities of all these QSAR models are compared by classical metrics and the best models are discussed in statistical and physicochemical terms.

## 1 Background and Motivation

At present, the pursuit of effective treatments for neurodegenerative disorders is one of the imperative clinical and social needs. The number of patients affected by those pathologies, including Alzheimer’s and Parkinson’s diseases, rise every year, principally in developed countries, directly related with the longer life expectancy. Parkinson’s Disease (PD) is the second most frequent human neurodegenerative disorder in persons over 60 years of age, affecting 1 in 100 people and growing to that affects 2–3% of the population ≥65 years of age. It is linked with Lewy bodies, abnormal aggregates of α-synuclein protein, and loss of dopaminergic neurons in the *substantia nigra*. Though clinical diagnosis is based on the presence of bradykinesia and other cardinal motor issues, Parkinson disorder is related with many non-motor symptoms that add to overall disability.

Genetic and epidemiological studies carried on numerous families in Asia, the United States, and Europe led to discover in 2004 that mutations in a new gene, known as leucine-rich repeat kinase 2 (LRRK2), are a key genetic risk factor for familiar and sporadic PD [1]. Nowadays, LRRK2 is one of the most pursuing and auspicious targets for the future pharmacological treatment of PD. In this regards, vast efforts are being done both from academia and pharmaceutical industry with the aim of designing selective and brain-permeable LRRK2 inhibitors as a strategy for PD [2], [3]. LRRK2 is an uncommon large protein (2527 amino acids) categorised as a member of the ROCO superfamily, which has a leucine-rich repeat (LRR) domain, a kinase domain, a DFG-like motif, a GTPase domain, a mixed lineage kinase (MLK) like domain, a RAS domain, and a WD40 domain. The protein is present mostly in the cytoplasm, though it is also connected to the mitochondrial outer membrane. The physiological role of LRRK2 is poorly understood and many of its substrates persist unclear. Nevertheless, it has been suggested to be helpful for preventing neurodegeneration [4], [5] and several LRRK2 inhibitors are being used as neuroprotective agents for PD. Additionally, several studies revealed that LRRK mutations raises aggregation of α-synuclein in dopaminergic neurons that are exposed to α-synuclein fibrils [6].

Quantitative structure–activity/property relationship (QSAR/QSPR) modelling has established itself as one of the main computational molecular modelling approaches, with a key role in drug virtual screening or optimization. QSAR models permit to identify relationships between structural information of chemical compounds (molecular descriptors) and a physicochemical or biological property under study. Nowadays, these methods are broadly used as a substitute for experimental studies to predict the activity of the molecules from their structure. In particular, machine learning techniques had become widely used in this field during the last decades [7].

Concerning scientific literature, a small number of QSAR studies in LRRK2 have been published. Furthermore, most of these works that have been presented, reported a very limited predictive activity for the external validation datasets [8], [9]. In this paper, novel QSAR models for predicting putative inhibitors of LRRK2 protein are inferred by using machine learning methods, extending the information and results reported in a conference article presented in the *12^th^ International Conference on Practical Applications of Computational Biology & Bioinformatics* [10]. In particular, several regression and classification QSAR models together with their accuracy performances, contrasted in terms of accuracy and model complexity, are discussed.

## 2 Materials and Methods

### 2.1 Ligand Preparation

Datasets on SMILES format were converted to 3D structures using LigPrep [11] software implemented on Maestro Suite [12]. LigPrep is a 2D-to-3D conversion tool that includes the addition of hydrogen atoms and options for generating multiple possible tautomers, stereoisomers, ionization at a selected pH range, and ring conformations using molecular mechanics force fields. To carry out our studies, possible ionizations were generated at pH 7.3 in order to obtain the most suitable ionization states of the compounds for physiological pH conditions. The ionization states were assigned with Epik module [13]. Also, all the compounds were desalted and no tautomers were generated. In this process, we have restricted the search to obtain just one possible stereoisomer among all that can be found by the program, as well as one low energy ring conformation. The final step of a LigPrep preparation is an energy minimization of the 3D conformers generated using the OPLS 2005 force field [14].

Different conformers and ionization states of the same compounds were reduced in order to keep one 3D structure per initial compound. The selection was made considering the most probable ionization state at physiological pH conditions. This preparation is a crucial step for the following studies and was performed with the aim of obtaining the most suitable 3D structures to further calculate the physicochemical properties of the existing compounds.

### 2.2 Drug-Like Properties Calculation

All the prepared compounds were analysed using Qikprop [15] module of the Small-Molecule Drug Discovery Suite in Schrödinger, an accurate software that predicts structurally significant 2D and 3D descriptors and pharmaceutically relevant properties of organic molecules. A total number of 44 properties could be predicted regarding Absorption, Distribution, Metabolism and Excretion (ADME). Among all the properties, the program calculates parameters such as molecular weight, QPlogPo/w (predicted octanol/water partition coefficient), molecular volume, number of H-bond donors or acceptors, polar surface area and violations related to the Lipinski’s Rule of 5 and Jorgensen’s Rule of 3. This allows filtering out compounds with clear cut undesirable properties for drug discovery.

### 2.3 Model Building

The first step consisted in dividing the dataset into a training (75%) and a test set (25%) following a stratified division. The next step is based on computing the molecular descriptors of all the dataset using DRAGON software [16]. DRAGON is an application tool that provides over 3000 diverse molecular descriptors (0D, 1D, 2D and 3D) which can be used to evaluate molecular QSAR or QSPR of different databases. To calculate these molecular descriptors, molecules were prepared as it is described in the ligand preparation section. After computing the descriptors, those descriptors that present over 0.95 correlations were discarded. Then, the next stage that takes place is the feature selection procedure and it is performed with DELPHOS [17]. This tool infers multiple alternative selections of molecular descriptors computed by DRAGON for defining a QSAR model. In this work, a total of 25 putative subsets have been computed, selecting the best 4 models in terms of MAE and MSE to be further selected to build QSAR models. In the case of the classification models, the target property was discretizated for defining classes using the following threshold: Low activity ≤50% > High activity.

Finally, in order to build the models, WEKA software [18] was used. This software is a collection of machine learning techniques for data mining. For each inference method, the parameter settings provided by default for WEKA were used in the experiments. The algorithms used in this study are described as follows:

Neural Networks (Multiperceptron): A classifier that uses back-propagation to classify instances. The network nodes are all sigmoid, excluding the cases where the class is numeric (in this situation the output nodes become unthresholded linear units).

Random Forest: class for constructing a forest of random trees. The random trees for constructing a tree that considers K randomly chosen attributes at each node. Also, it has an option to allow estimation of class probabilities (or target mean in the regression case) based on a hold-out set (back fitting).

Random Committee: class for building an ensemble of randomized base classifiers. Each base classifier is built using a different random number of seed (but based on the same data). The final output is a straight average of the predictions obtained by the individual base classifiers.

Metrics of the best models are reported in the work and the descriptors present in the best models are analysed in terms of correlation using VIDEAN tool.

## 3 Results and Discussion

**Figure 1:**
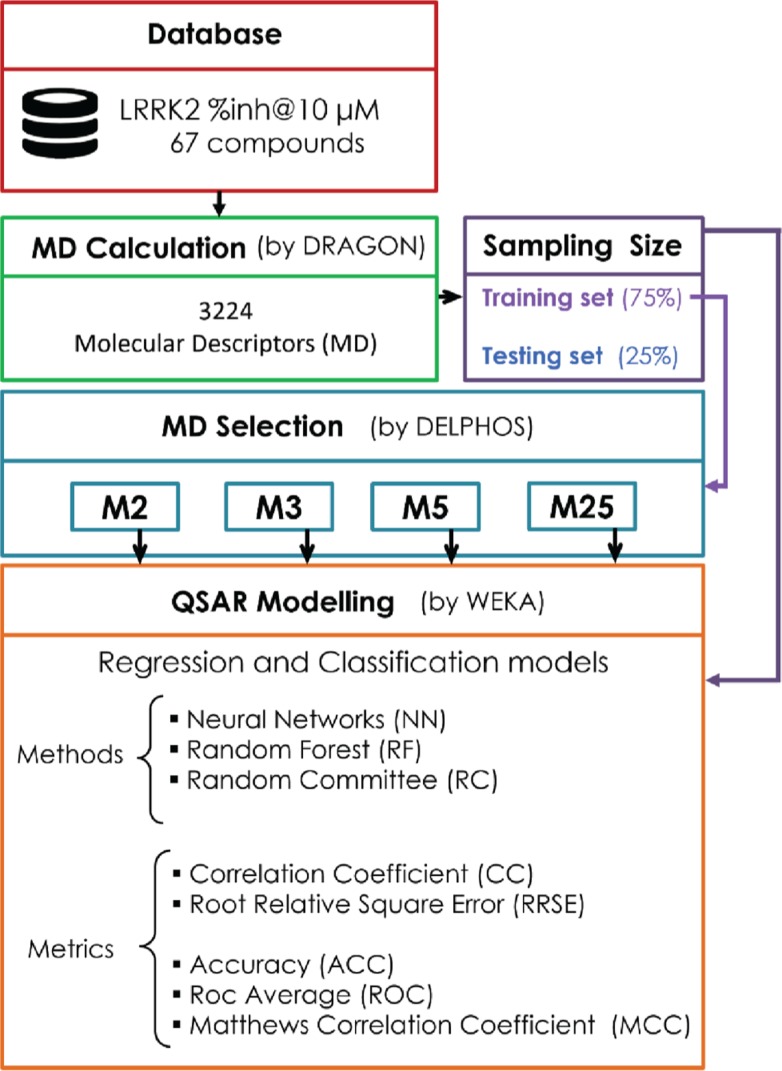
Methodology carried out for generate the different QSAR models.

QSAR models, both for classification and regression models, learned by a multi-objective feature selection technique and machine learning methods are described. Figure 1 presents an outline of the experimental process followed in this work. The database is a compilation of 67 molecules formerly synthesized in our research group and tested in LRRK2 enzyme [19]. In this assay LRRK2 kinase activity was measured and the results expressed as the percentage of enzyme inhibition for every compound, as it is further discussed in the previous reference. Table 1 presents this information. A central step in QSAR studies is to collect a representative set of compounds in order to comprise a diversity physicochemical space. With the goal of scrutinising this dataset, we have carried out a characterization of the molecules from a physicochemical perspective and a drug-like point of view. Physicochemical and drug-like properties of this dataset were computed using Qikprop application and the most representative descriptors were analysed to show the diversity of the dataset.

**Table 1:** Structure of the chemical compounds in the database and their percentages of enzymatic inhibition.

Structure	LRRK2 %inh @10 μM CI_50_ (μM)	Structure	LRRK2 %inh @10 μM CI_50_ (μM)	Structure	LRRK2 %inh @10 μM CI_50_ (μM)	Structure	LRRK2 %inh @10 μM CI_50_ (μM)
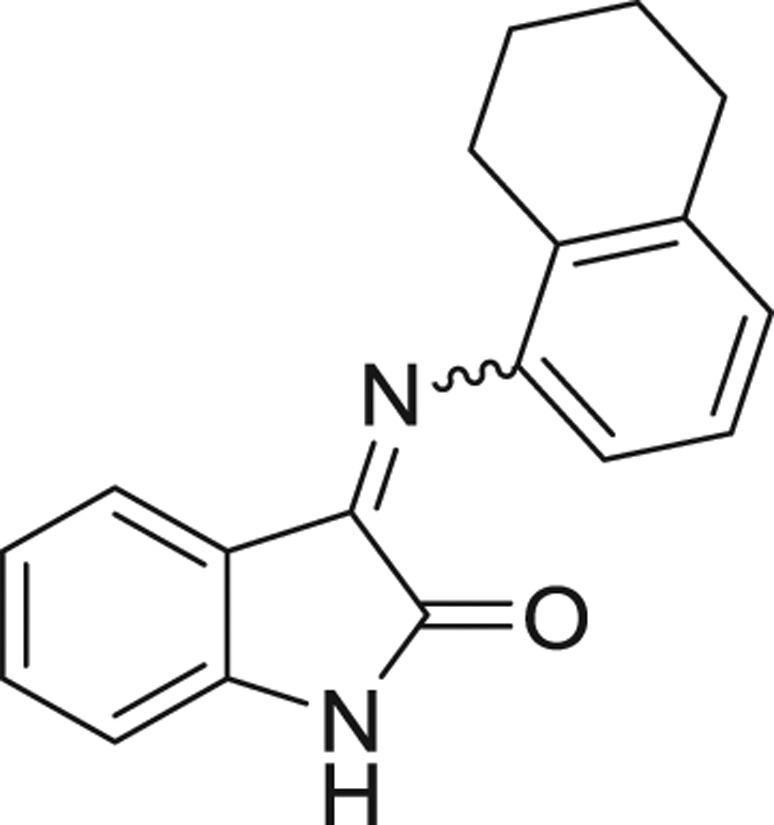	14%	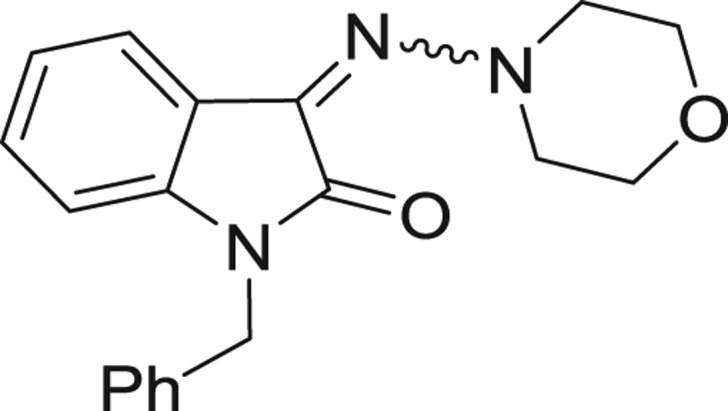	2%	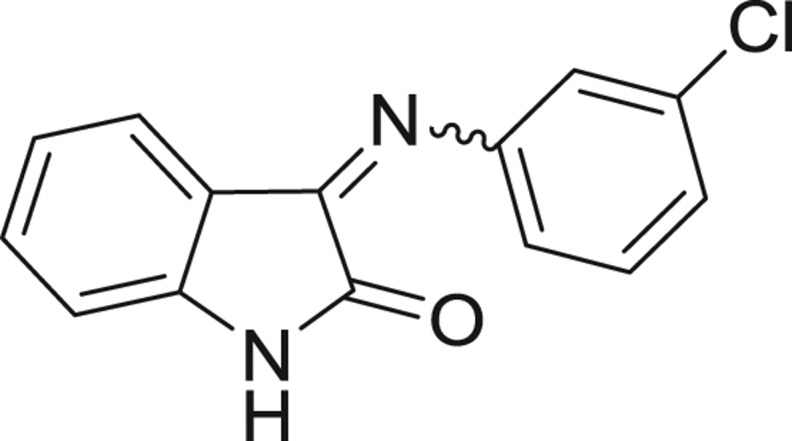	39%	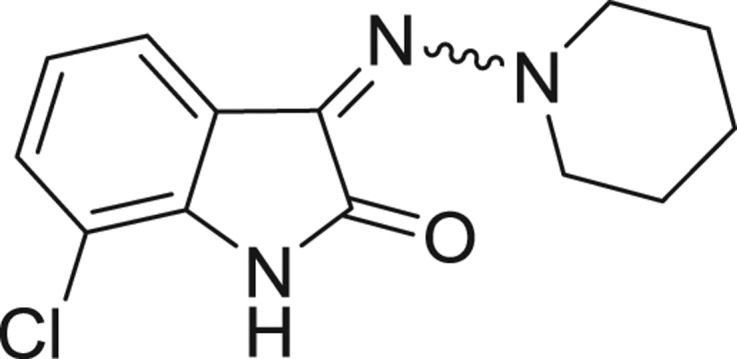	11%
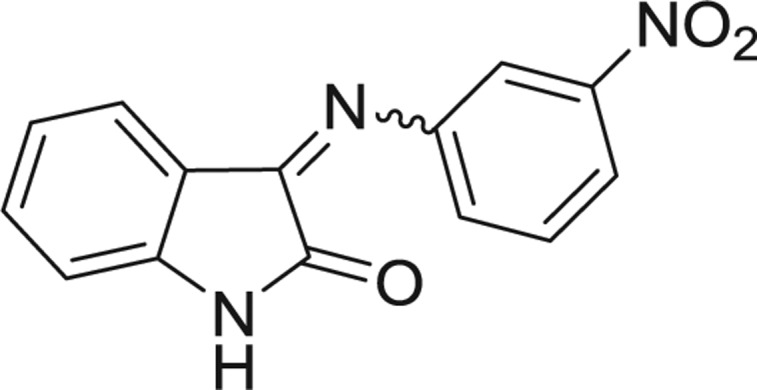	19%	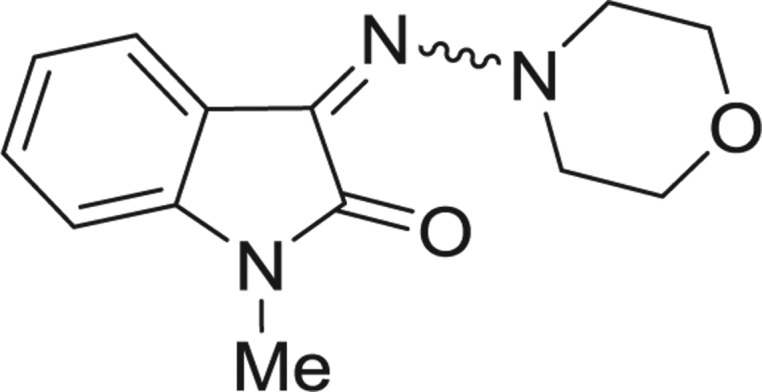	6%	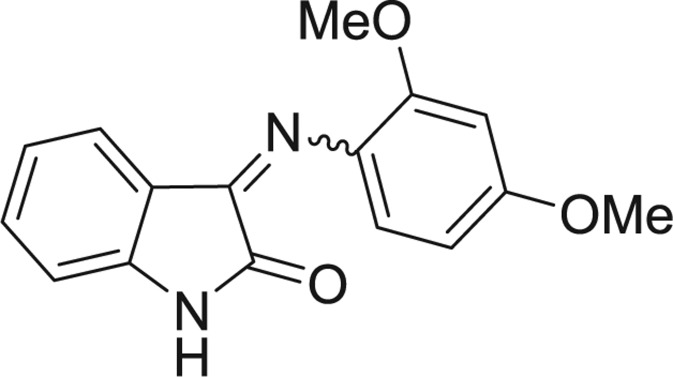	15%	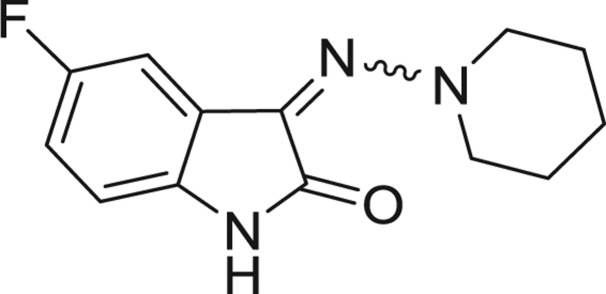	77%
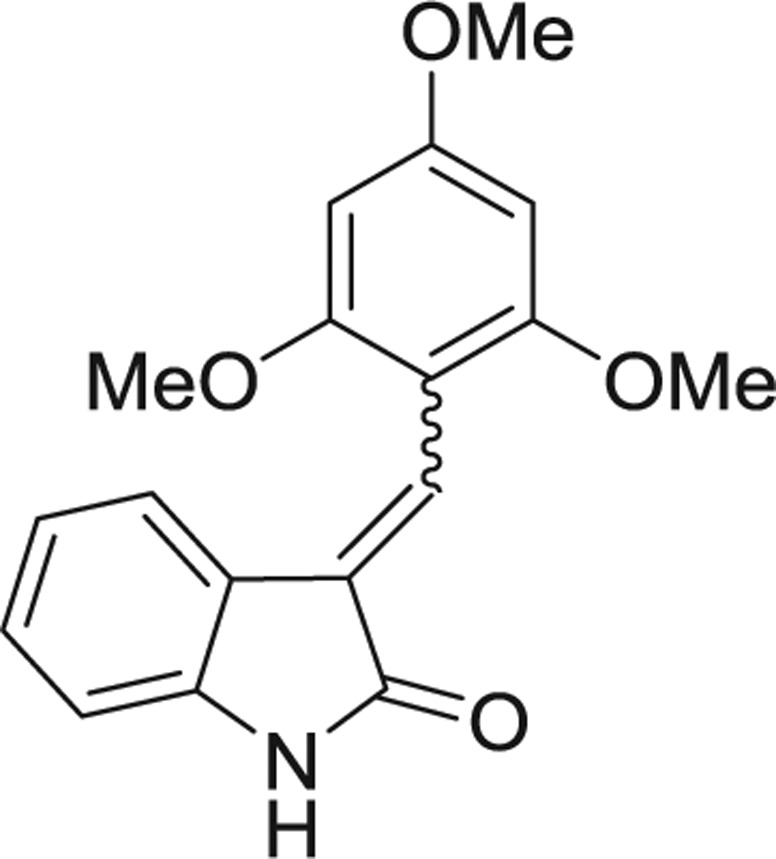	0%	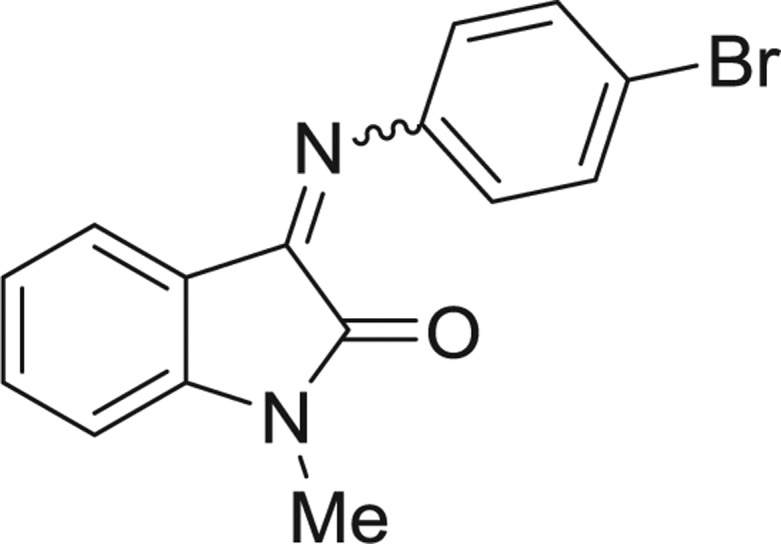	9%	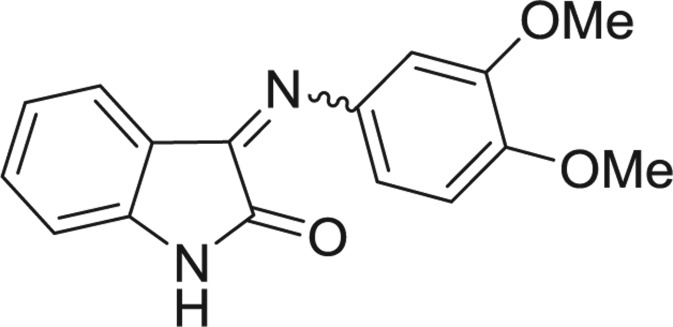	3%	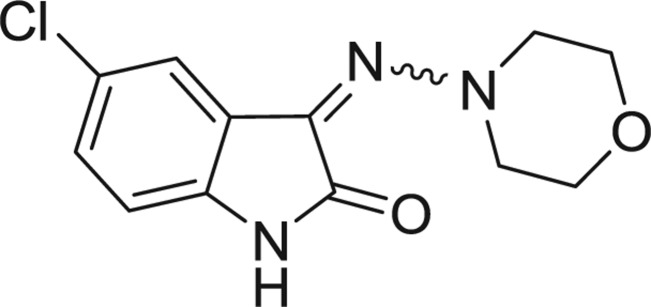	78%
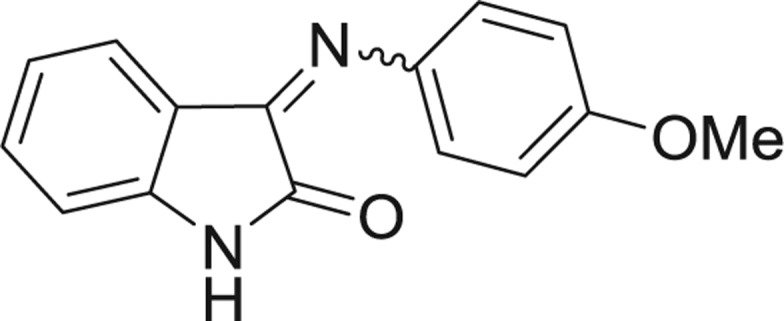	63%	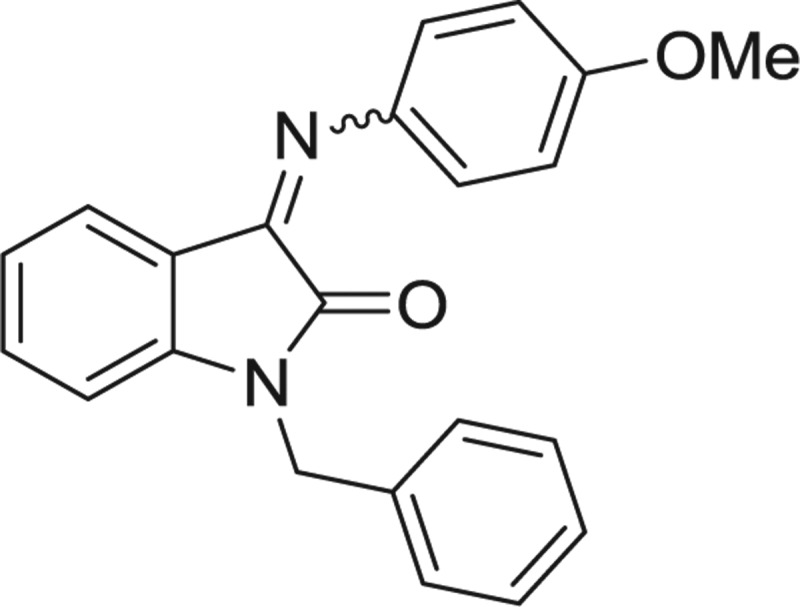	13%	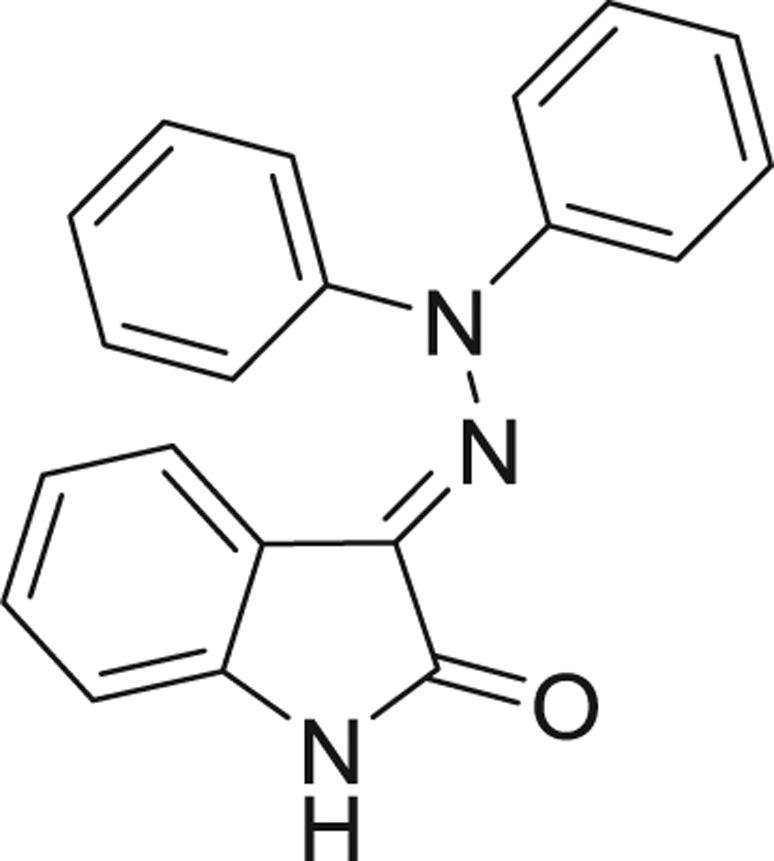	96%	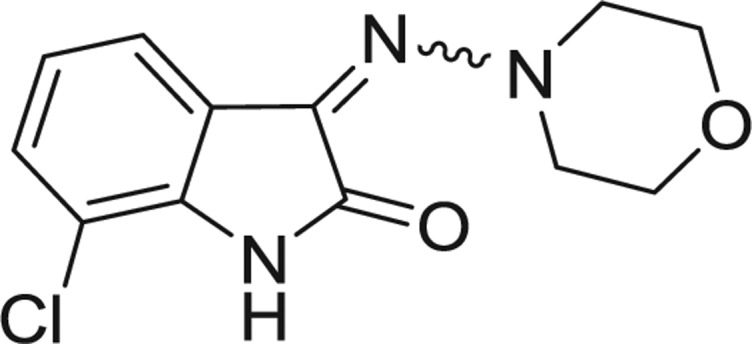	7%
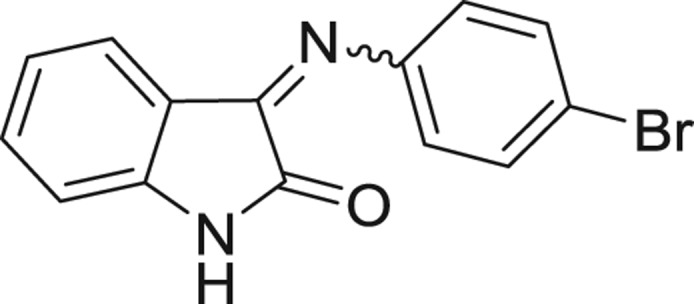	11%	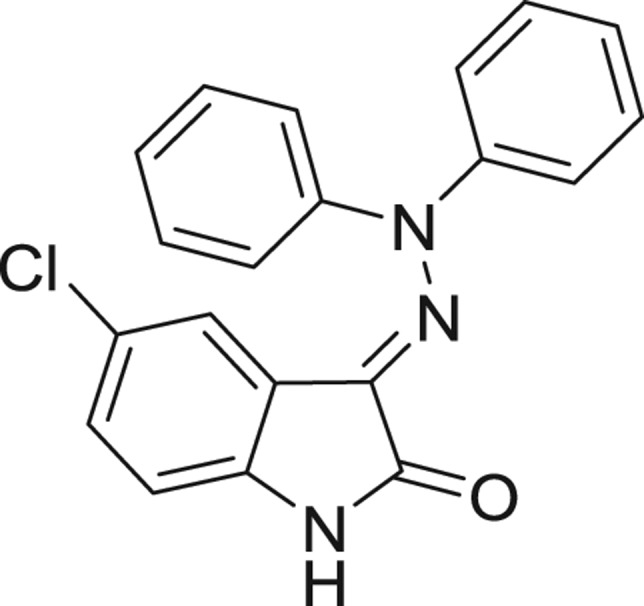	96%	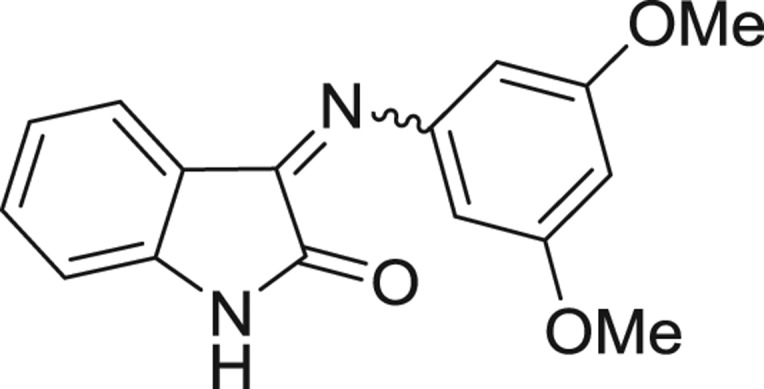	7%	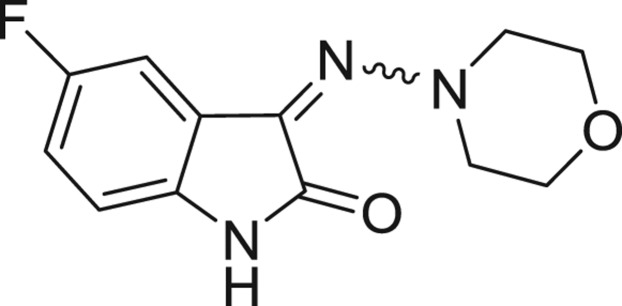	66%
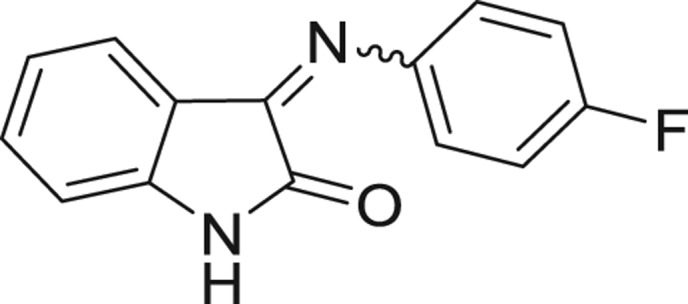	71%	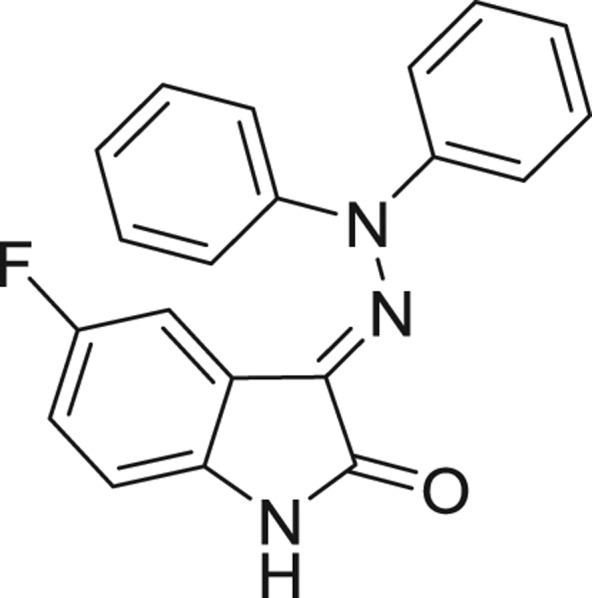	97%	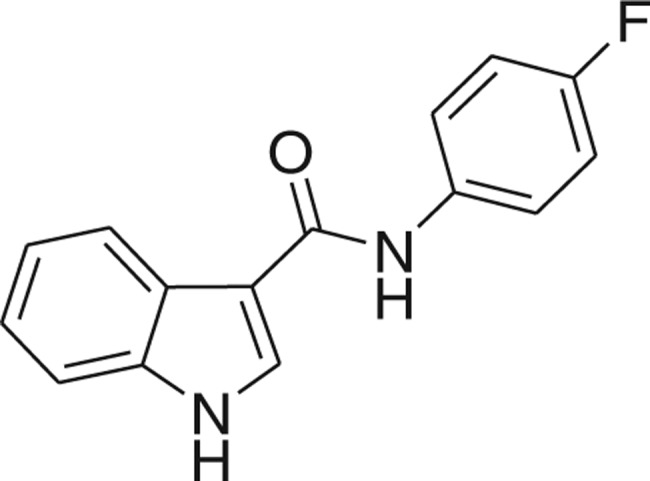	23%	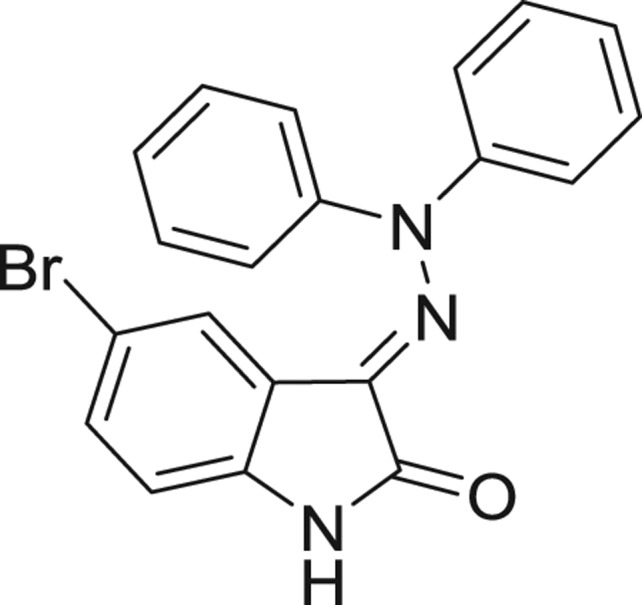	91%
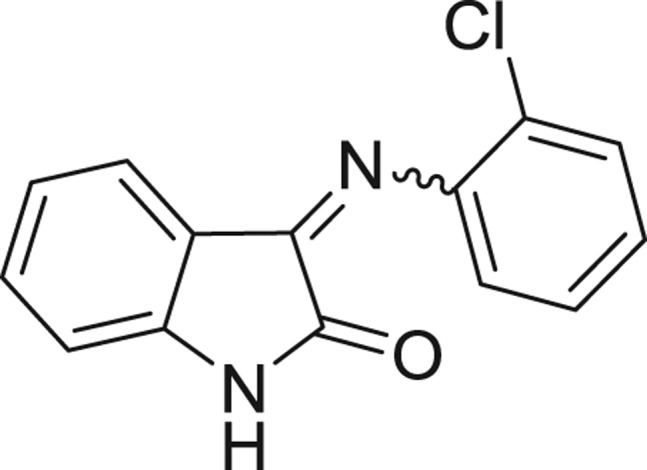	30%	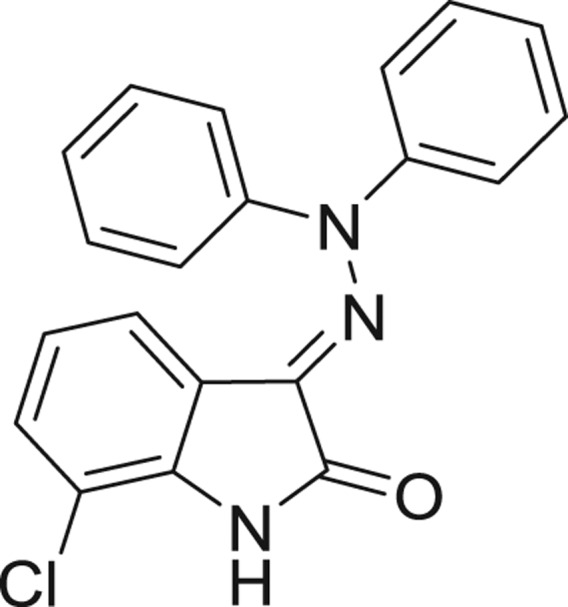	17%	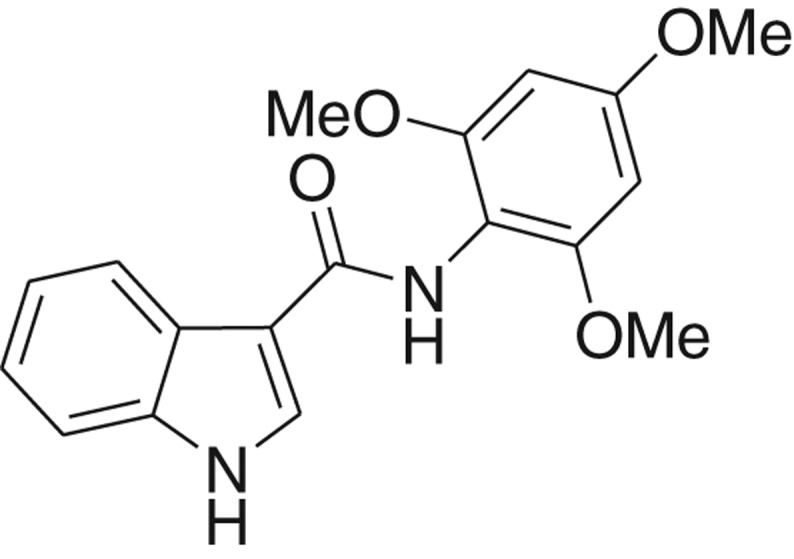	0%	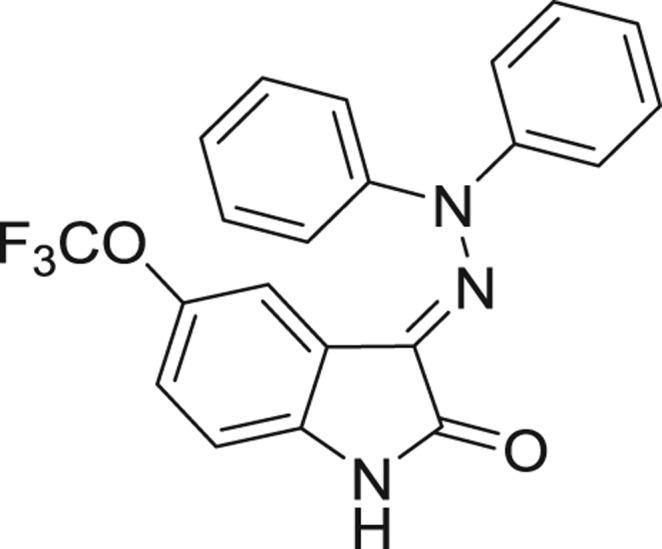	61%
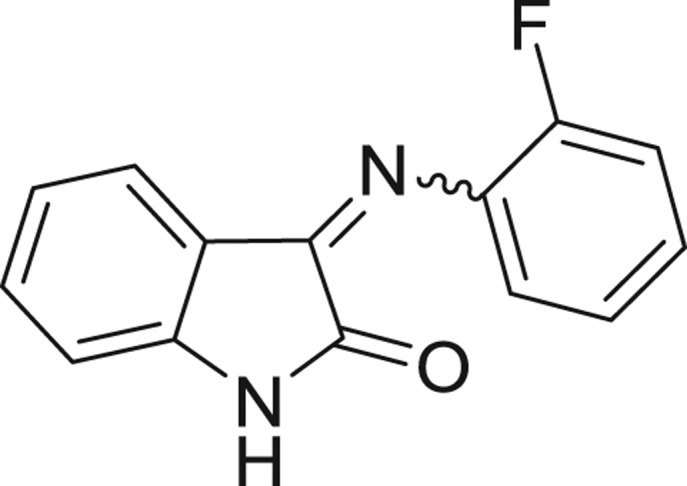	60%	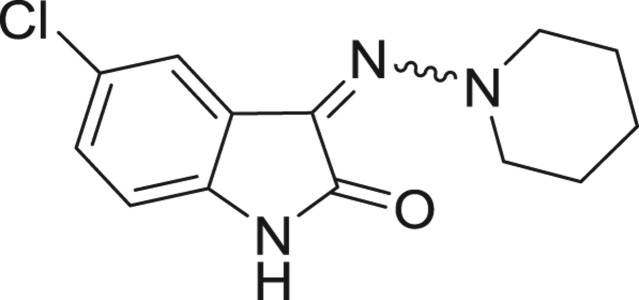	91%	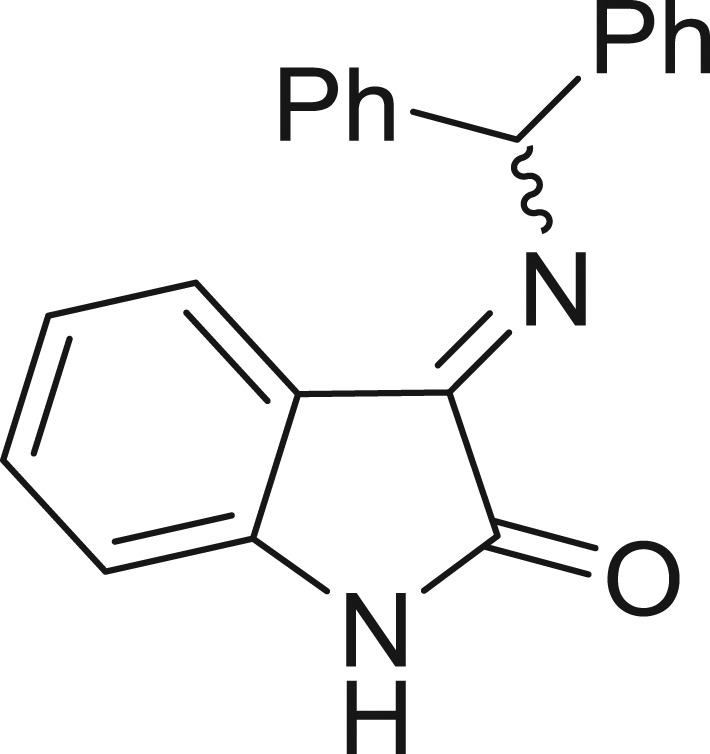	0%	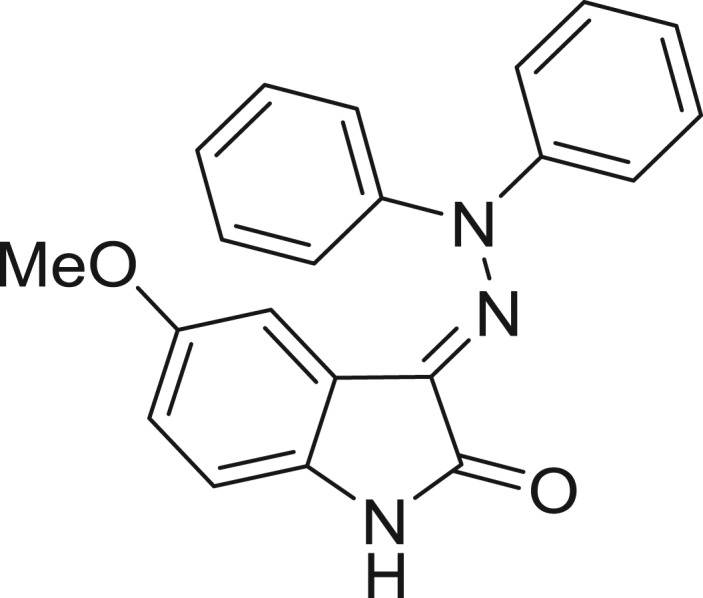	100%
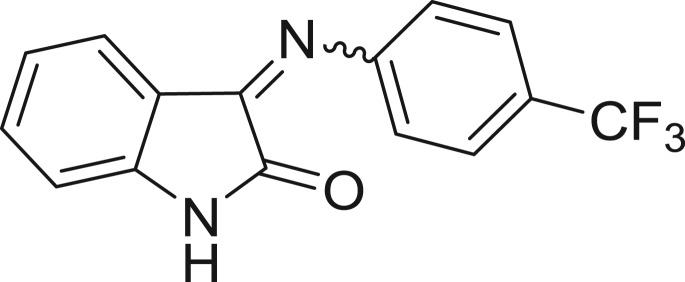	14%	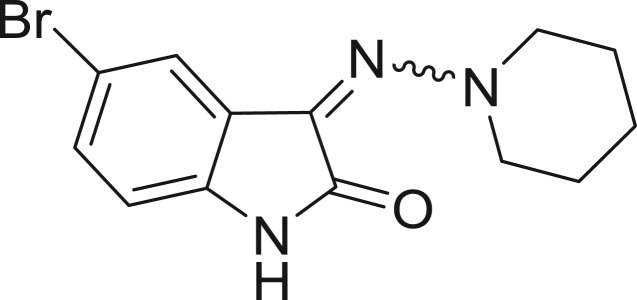	92%	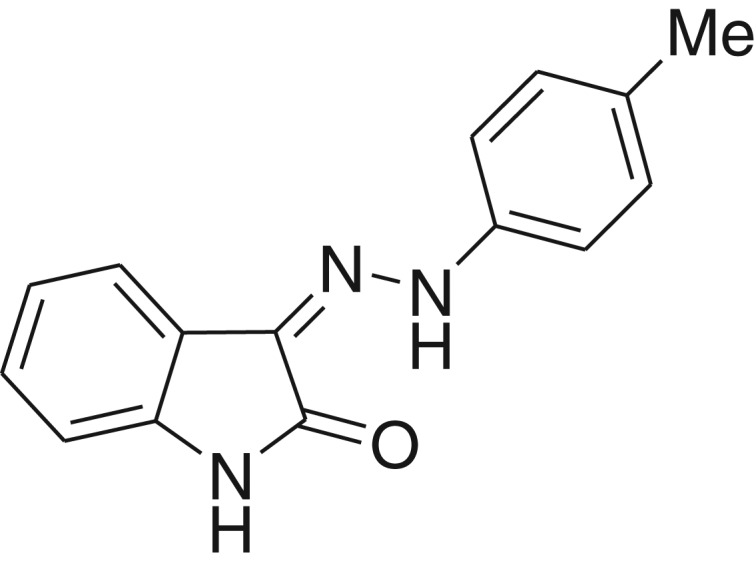	23%	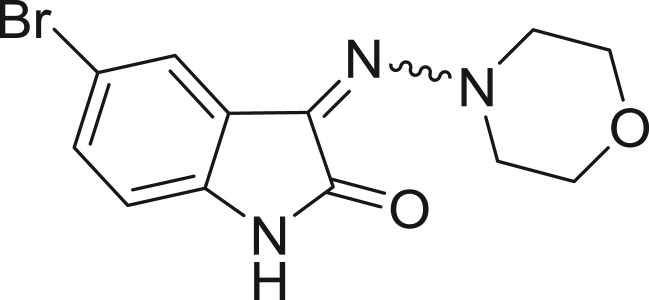	91%
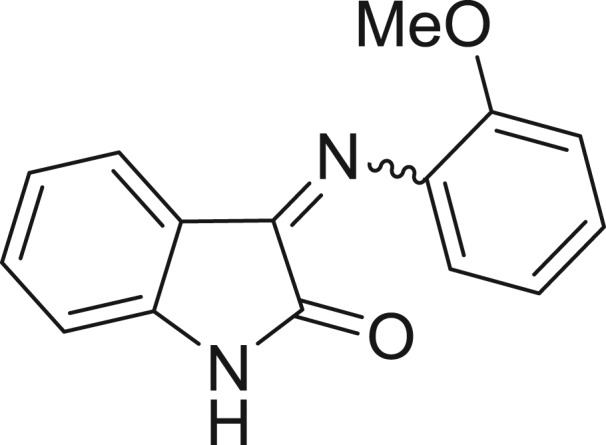	15%	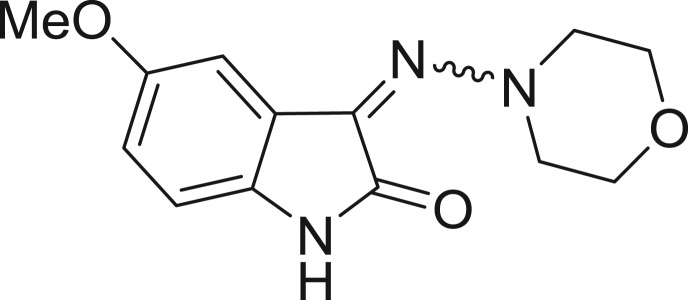	72%	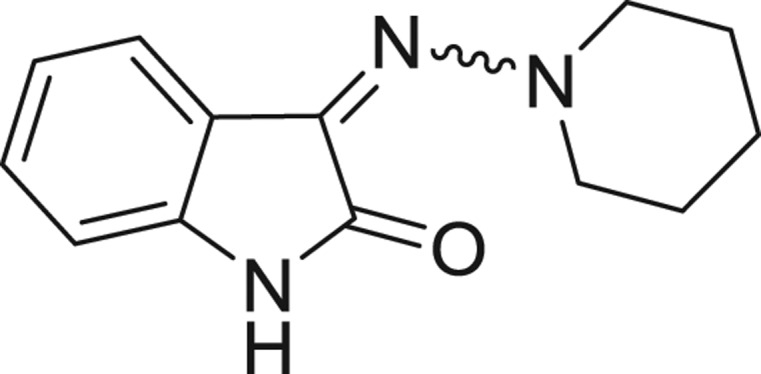	79%	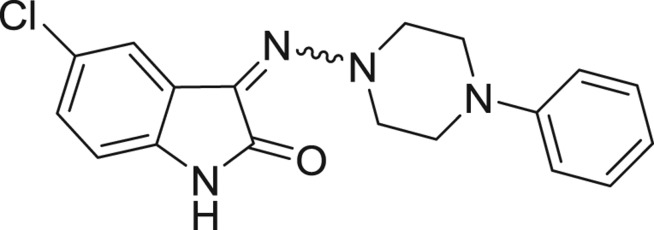	45%
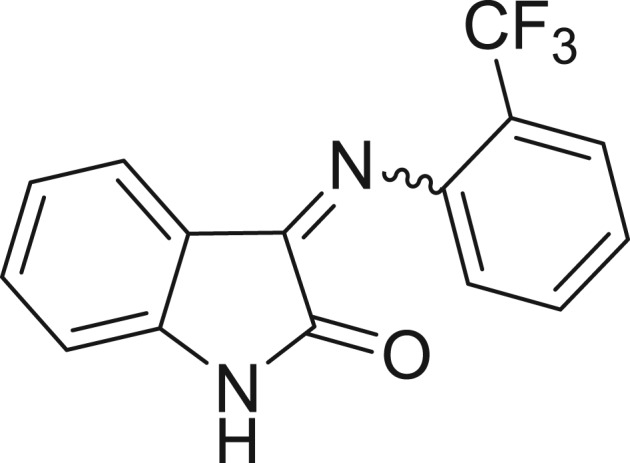	12%	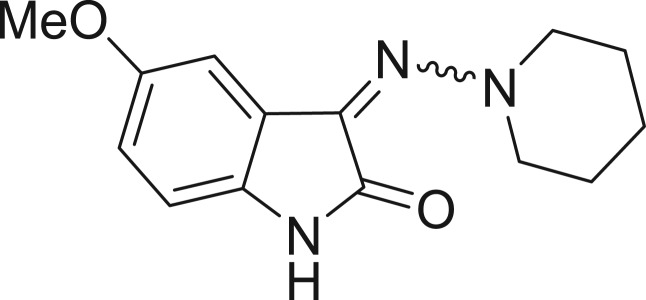	94%	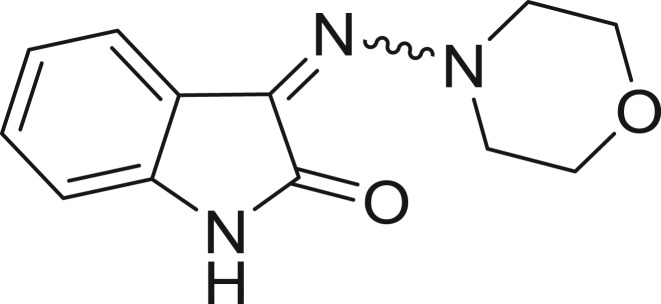	74%	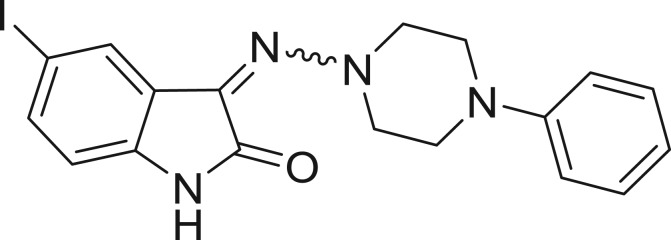	65%
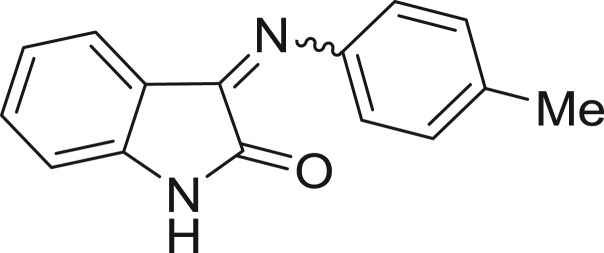	54%	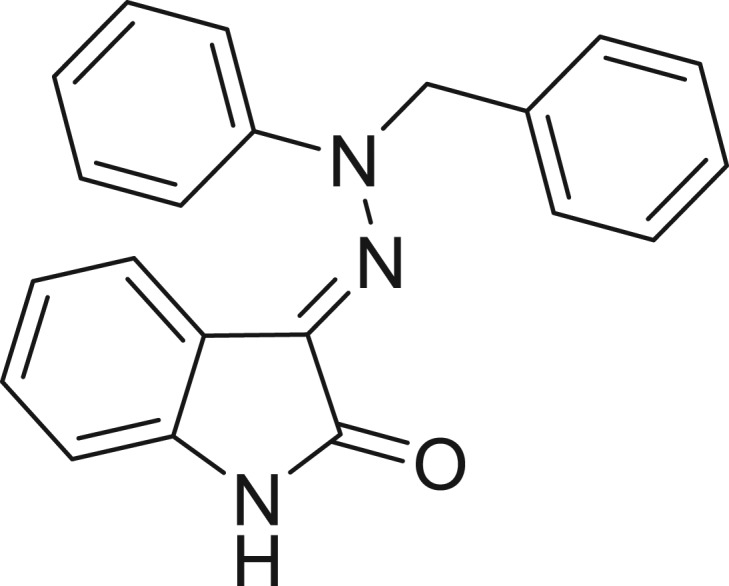	71%	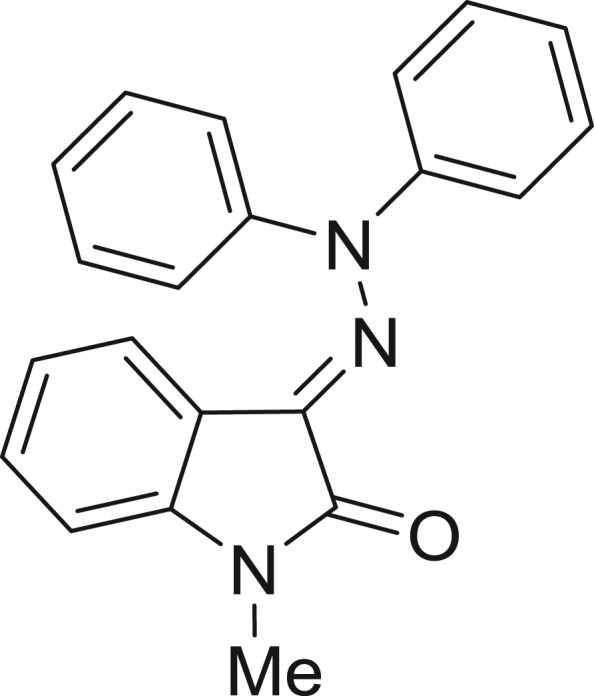	49%	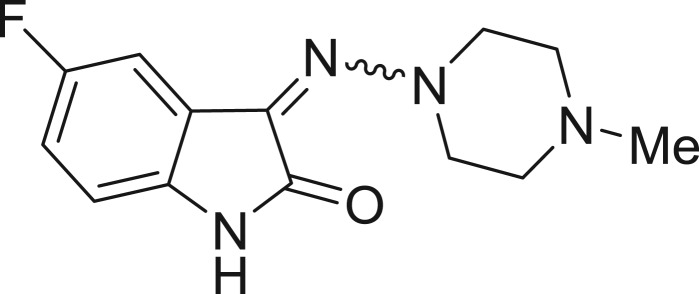	11%
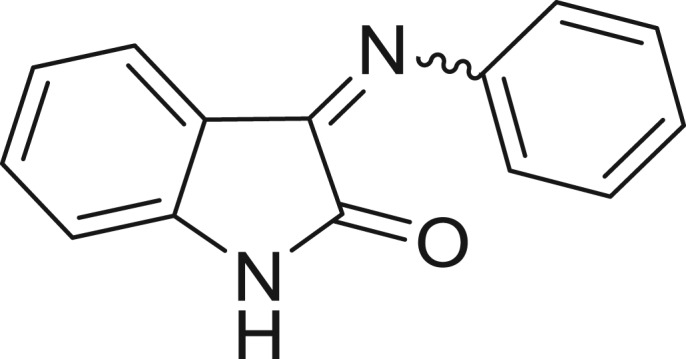	71%	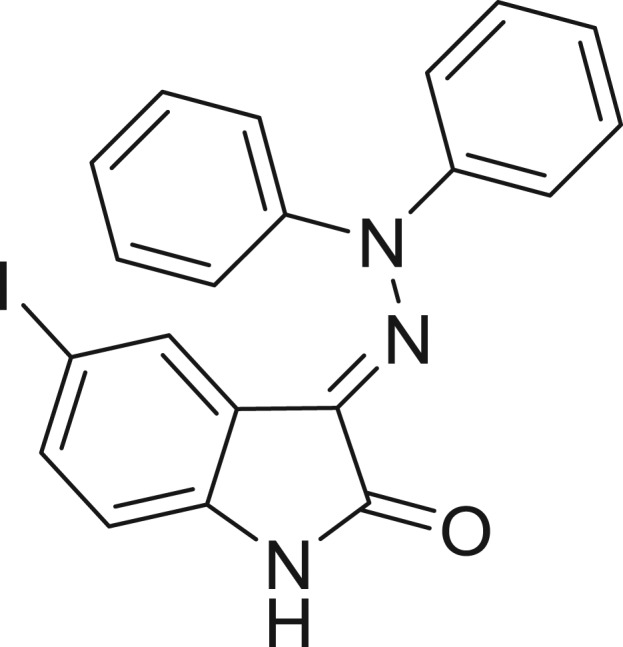	99%	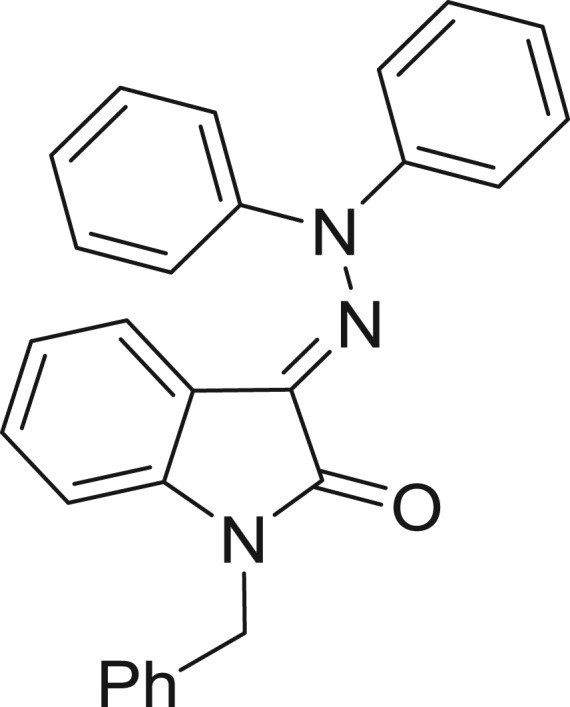	40%	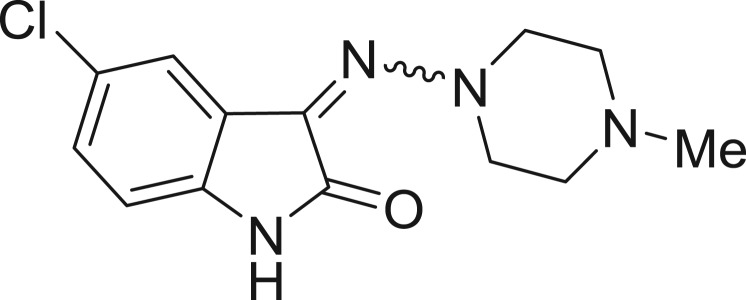	36%
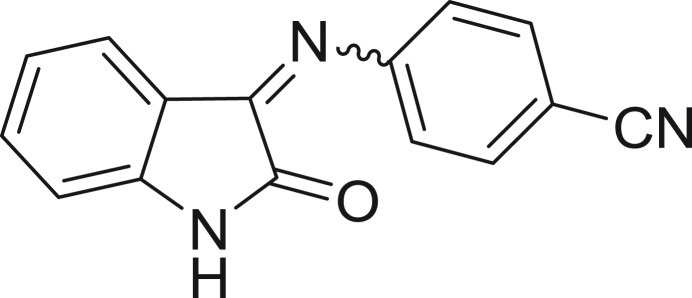	45%	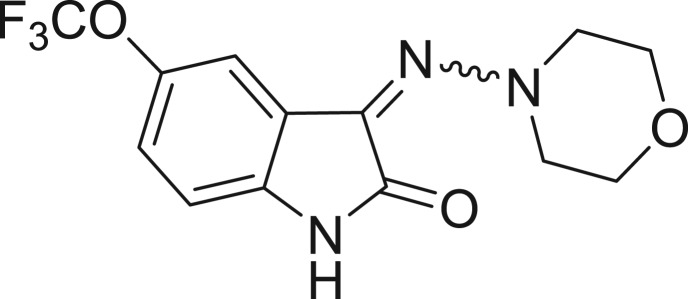	54%	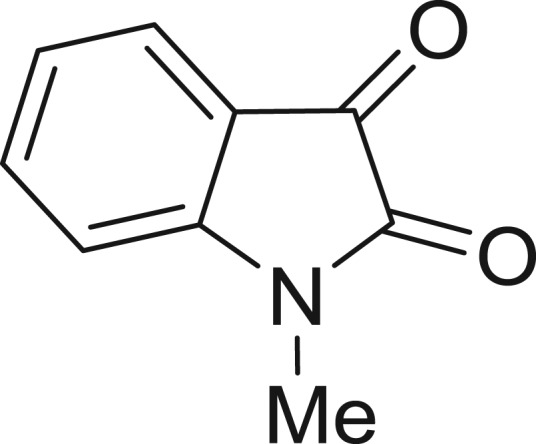	0%	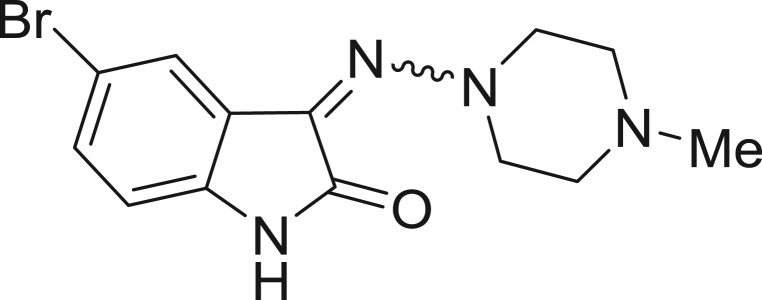	35%
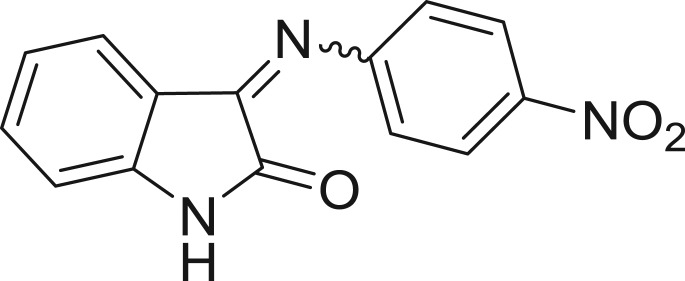	66%	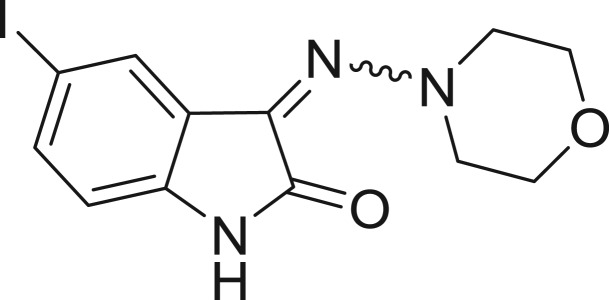	95%	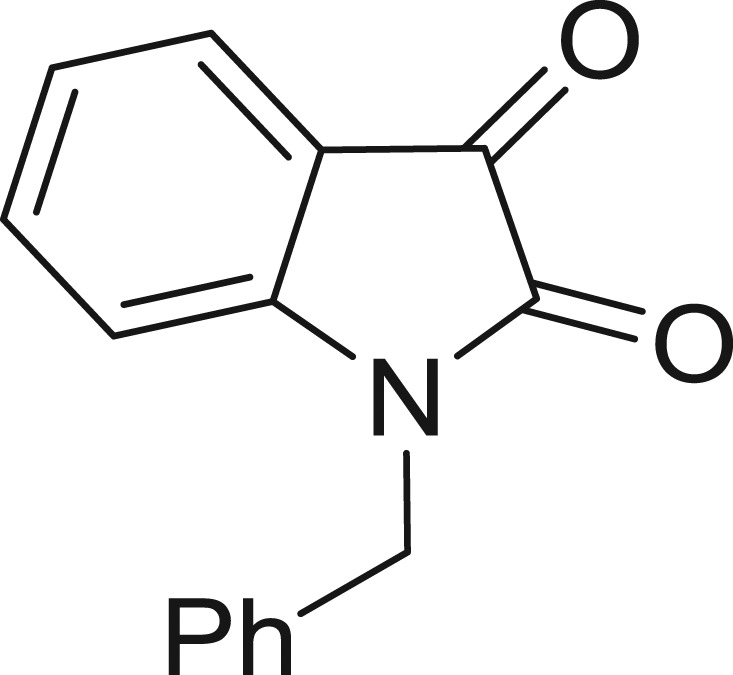	0%	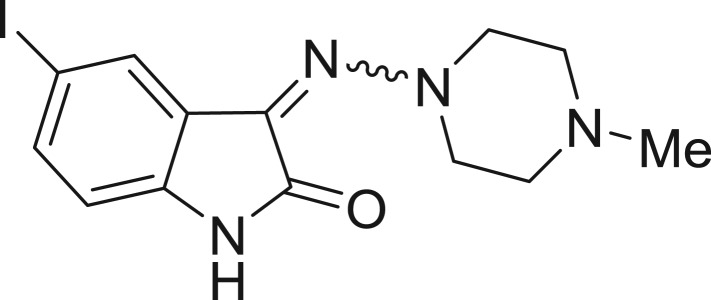	62%
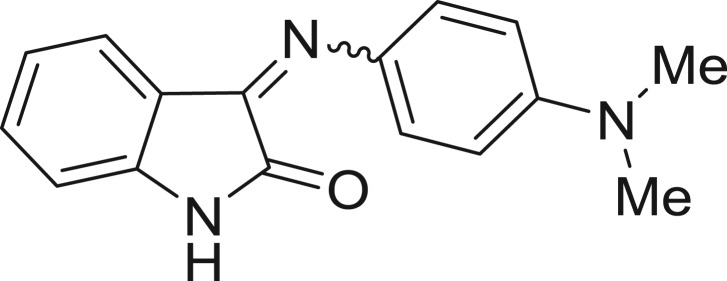	76%	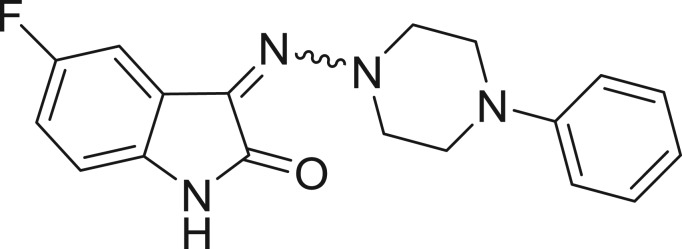	39%	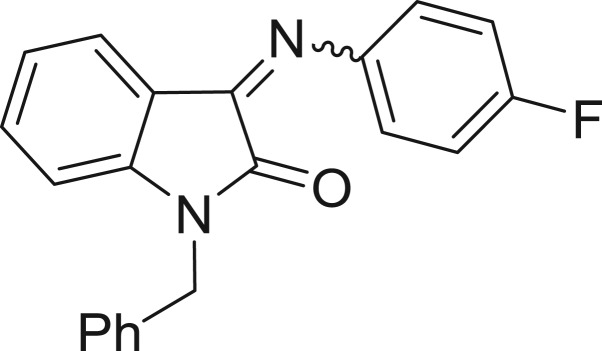	1%	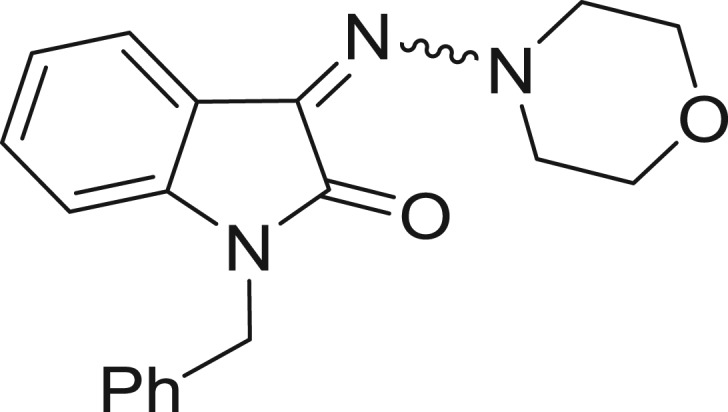	2%
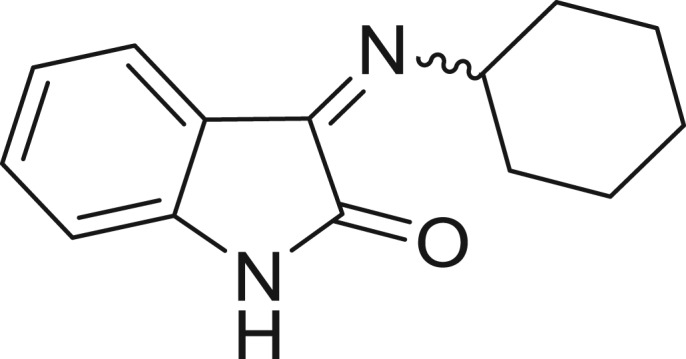	36%	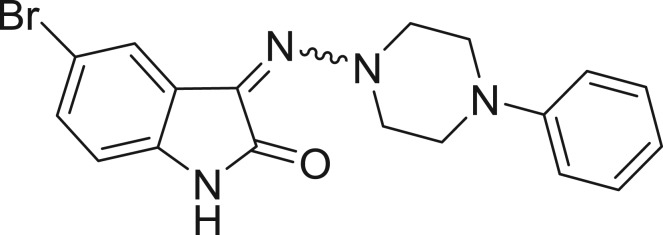	51%	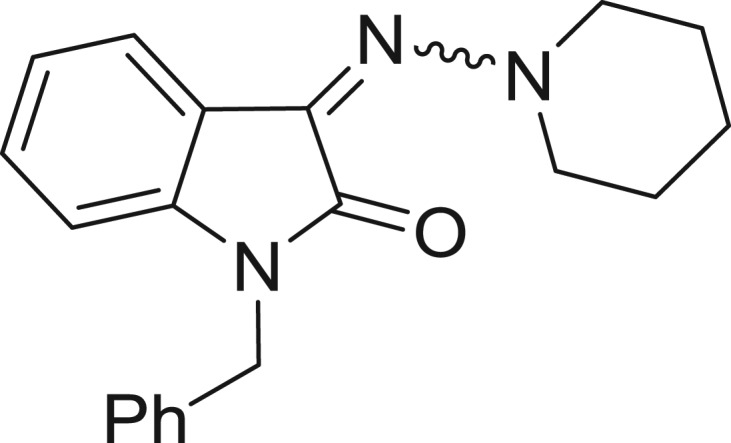	1%		

**Figure 2:**
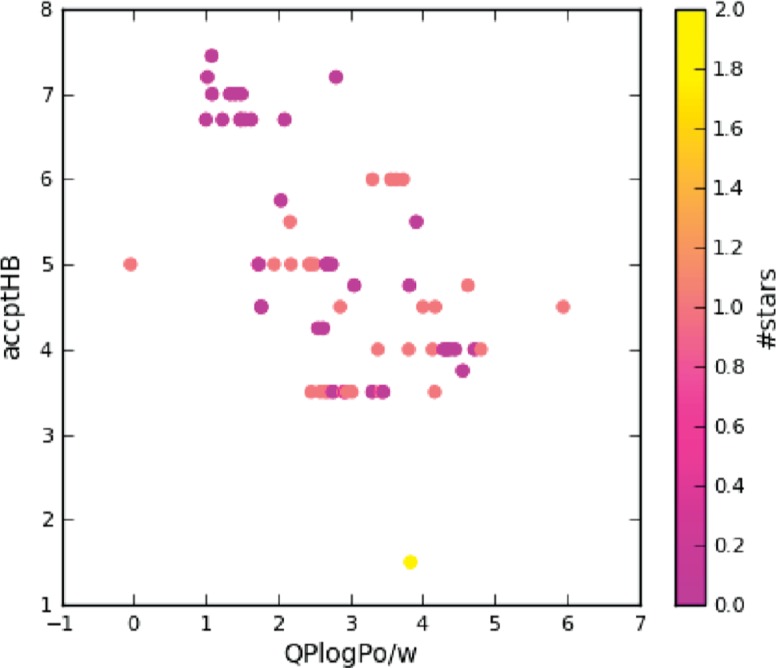
Dispersion of the database molecules taking into account some of the QP properties calculated such as logP values and number of acceptor H-bonds. Color scale is defined by the stars values.

Some of these relevant parameters are depicted in Figure 2, where we can note a wide dispersion of two different key properties in drug discovery such as logP (x axis) and H-bond acceptors (accptHB y-axis). Molecules are coloured taking into account their stars values. This parameter represents the quantity of properties or descriptors values computed that fall outside the 95% range of similar values for known drugs. For this reason, a large value of stars indicates that a molecule present less drug-like properties. In our case, all compounds present values that are equal or lower than 2, which means that the complete database consists of drug-like structures. Moreover, considering Lipinski rule of 5 [20], the 67 molecules present 1 or none violations of the rules, which means that the compounds are likely orally active drugs in humans. Consequently, and subsequently with this analysis, we can determine that the database is diverse in terms of physicochemical properties and all the molecules are drug like.

### 3.1 QSAR Models

A total quantity of 3224 descriptors were calculated using Dragon for the complete database. The experiments were executed following the rationale schematized in Figure 1. The dataset was split into training set (75%) and test set (25%) using a stratified sampling. Several alternative subsets of descriptors were selected from the training set using DELPHOS [17]. This software repeats 10 times the random splitting of the data in 75/25 in order to perform 10-cross-fold validation of the selected subsets. In Table 2, a summary of the molecular descriptors subsets with lower relative absolute error (RAE) values is reported. Using these four subsets and different inference techniques, a variety of QSAR regression and classification models were generated. The models are calculated by WEKA [18] using Neural Networks (NN), Random Forest (RF) and Random Committee (RC) as learning methods, where default parameters provided by WEKA were used for calculating the models. The decision of applying several methods for QSAR modelling is due to the fact that recent studies have argued that it does not exist a more advisable strategy for QSAR learning from the subsets of descriptors [21], [22]. For classification models, the discretization thresholds of the target property values were: low activity ≤50% and high activity >50%.

**Table 2:** Best molecular descriptors (MDs) subsets obtained by DELPHOS in the feature selection process.

Subset	MDs		Descriptor Type	Size
M2	MW		Constitutional indices	4
	MWC08		Walk and path counts	
	BEHp2		Burden eigenvalues	
	RDF105p		RDF descriptors	
M3	MW		Constitutional indices	4
	JGI2		2D autocorrelation	
	HATs6m	R2e	GETAWAY descriptors	
M5	MW		Constitutional indices	5
	IC0		Information indices	
	ESpm09x		Edge adjacency indices	
	JGI3		2D autocorrelation	
	L3s		WHIM descriptors	
M25	MW		Constitutional indices	13
	HNar	ECC	Topological indices	
	GATs7e		2D autocorrelations	
	VEZ1	VEp2	2Dmatrix-based descriptors	
	DISPm		Geometrical descriptors	
	RDF105p		RDF descriptors	
	R8e		GETAWAY descriptors	
	B06[N-Br]	B07[C-Cl]	2D atom pairs	
	F04[C-C]	F05[O-Cl]	2D atom pairs	

Table 3 displays performance metrics reported by WEKA for the best regression and classification QSAR models obtained using each descriptor subset. The results for classification models are detailed using the accuracy (ACC), namely percentage of cases correctly classified, the average Receiver Operating Characteristic (ROC) and the Matthews Correlation Coefficient (MCC). For regression models, the correlation coefficient (CC) and the root relative square error (RRSE) results are presented.

**Table 3:** Predictive accuracy of the best classification and regression QSAR models evaluated over the testing set.

Model	Size	Best Classification QSAR Models				Best Regression QSAR Models		
		Method	ACC	ROC	MCC	Method	CC	RRSE
M2	4	RF	68.8	0.69	0.40	RF	0.55	87.50
M3	4	*RC*	*87.5*	*0.91*	*0.77*	RC	0.68	74.69
M5	5	RC	75.0	0.95	0.52	*RF*	*0.83*	*60.82*
M25	13	RC	75.0	0.73	0.53	RC	0.44	92.34

The classification model with higher performance was inferred from the subset M3 by using Random Committee and achieved 87.5% of correct classified samples with a ROC value of 0.91. These results are shown in Table 3. On the other hand, the best regression model was inferred with the subset M5 by using Random Forest and obtained a correlation coefficient of 0.83 and a RRSE of 60.82. The datasets used for learning are available at: http://lidecc.cs.uns.edu.ar/pacbb2018.LRRK2/datasets.html.

The best model inferred for the regression case has five molecular descriptors ranging a wide variety of different descriptors classes, from 0D molecular descriptors to 3D. For example, molecular weight is a constitutional index, belonging to the class of 0D-descriptors, that is obtained from the chemical formula, as they do not consider the tridimensional structure of the ligands. We have also found ESpm09x descriptor, spectral moment 09 from edge adjacency matrix weighted by edge degrees and IC0 information content index (neighbourhood symmetry of 0-order) and JGI3: mean topological charge index of order 3 that are 2D descriptors from different families. Lastly, the 3D descriptor found in this model is the L3s: 3^rd^ component size directional WHIM index/weighted by I-state.

Concerning the best classification model, includes four descriptors and we have also selected a wide representation of different descriptors families. MW has also been included in this model as a 0D descriptor. A very related descriptor to JGI3 was also used in this model, JGI2, is a 2D descriptor and is related to the charge index of the compounds in the database. To conclude, two 3D descriptors were chosen by DELPHOS that are R2e: R autocorrelation of lag 2/weighted by Sanderson electronegativity andHATs6m: leverage-weighted autocorrelation of lag 6/weighted by mass. Both are GETAWAY descriptors (GEometry, Topology, and Atom-Weights AssemblY) and are related to electronegativity and mass, key parameters in protein-ligand recognition process for protein inhibition.

**Figure 3:**
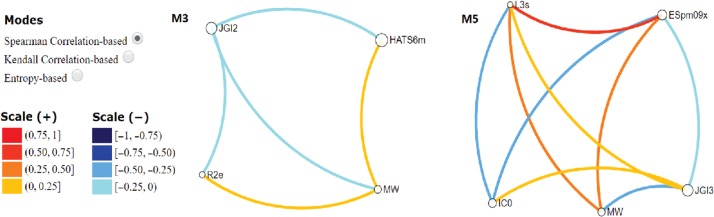
Kendall correlation grade between the descriptors of M3 y M5 subsets.

Additionally, we studied the relationship among the descriptors in statistical terms by using VIDEAN software, which is a visual analytics tool for the analysis of molecular descriptor subsets. It displays the associations and interactions among the molecular descriptors and the target property in statistical terms. The analysis of the pair correlation among the five descriptors that conform the regression model and the four descriptors that are part of the best classification model is presented in Figure 3 using Kendall correlation.

In this kind of analysis, the central aim is to detect models with low correlation among descriptors that means low redundant information. In this case, we perceive a clear majority of light tones of links, both blue and orange, that make connection between the descriptors (nodes) present in the models. This fact reveals the low data redundancy in both models that have been selected as the best ones.

## 4 Conclusions

Parkinson’s disease is one of the neurodegenerative illnesses with higher impact in elder people around the world. A promising target for its pharmacological treatment is the leucine-rich repeat kinase 2 (LRRK2). Several studies suggest LRRK2 inhibitors can play a key role for preventing neurodegeneration. For this reason, in this paper, QSAR models for virtual screening of putative inhibitors of LRRK2 protein has been designed. These models were obtained by machine learning methods over data from an in-house chemical library.

The computational approach used in this paper has two main steps: initially, alternative subsets of molecular descriptors relevant for structural characterization of LRRK2 inhibitors are identified by a multi-objective feature selection method; after that, several QSAR models are learned from these subsets by applying different supervised learning algorithms. The performance of these QSAR models was contrasted by classical metrics.

The molecular descriptor subsets related with the regression and classification models that yield the best performances were examined in statistical and physicochemical terms. From the analysis, it is possible to observe that the selected subsets have low cardinality but cover a wide spectrum of the molecular descriptor classes, contributing in this way with meaningful and diverse structural information to the models. Additionally, the visual analytics study reveals that the selected molecular descriptors offer non-redundant information in statistical terms.

Nonetheless, even when these QSAR models reach high accuracies, it is important to note that these models were learned from datasets integrated by a reduced quantity of molecules, which can limit the generalization properties of these QSAR models. For this reason, our piece of advice for the potential users of these models is to employ applicability domain methods over their testing compounds before using these models. As future work, we hope to extend the in-house chemical library for LRKK2 in order to improve the generalizability of these findings.
